# Peri‐Liver Transplant Hyperglycemia: Mechanisms, Associated Factors, Consequences, and Management – A Systematic Review

**DOI:** 10.1002/edm2.70107

**Published:** 2025-09-10

**Authors:** Yekta Rameshi, Simin Dashti‐Khavidaki, Soghra Rabizadeh, Mahta Alimadadi, Alimohammad Moradi, Amir Kasraianfard, Ali Jafarian, Zahra Ahmadinejad

**Affiliations:** ^1^ Department of Clinical Pharmacy, Faculty of Pharmacy Tehran University of Medical Sciences Tehran Iran; ^2^ Liver Transplantation Research Center Tehran University of Medical Sciences Tehran Iran; ^3^ Endocrinology and Metabolism Research Center (EMRC), Vali‐Asr Hospital Tehran University of Medical Sciences Tehran Iran

**Keywords:** glycemic control, hyperglycemia, liver transplantation

## Abstract

**Introduction:**

Liver transplantation is associated with various metabolic disorders. Peri‐transplant hyperglycemia is among the most frequent metabolic disorders among liver transplant recipients. Hyperglycemia following liver transplantation can increase the risk of post‐transplant complications, potentially impacting both graft and recipient outcomes. Several studies have compared intensive with standard blood glucose control strategies in liver transplant recipients. However, a comprehensive protocol for managing peri‐transplant hyperglycemia remains elusive. This review aimed to synthesise existing literature on the mechanisms, associated factors, and consequences of hyperglycemia after liver transplantation, and to provide recommendations for managing hyperglycemia in this patient population.

**Method:**

PubMed, Scopus, and UpToDate databases and American Diabetes Association guidelines were searched without time limitations until February 2025.

**Results:**

Peri‐liver transplant hyperglycemia can be attributed to several factors, including post‐reperfusion hepatocyte injury, insulin resistance stemming from underlying liver disease, surgical stress, and the use of immunosuppressive drugs. Various factors associated with peri‐transplant hyperglycemia can be categorised into pre‐transplant recipient factors, intraoperative factors, and donor‐related factors. Research has shown that inadequate glycemic control during the peri‐transplant period may have detrimental effects on post‐transplant outcomes, including an increased incidence of infections, graft rejection, acute kidney injury, prolonged hospital stays, and higher overall mortality.

**Conclusion:**

The suggestions presented in this article, which consider the recipient's medical history and clinical conditions, can serve as a framework for healthcare providers to manage peri‐liver transplant hyperglycemia effectively.

## Graphical abstract

Peri‐liver transplant hyperglycemia results from ischemia–reperfusion injury, immunosuppressive therapy, and surgical stress and may be influenced by some recipients, intraoperative, and donor factors. Possible associations between peri‐liver transplant hyperglycemia and some outcomes such as infections, graft rejection, and overall mortality emphasise the need for effective and individualised glycemic management of liver transplant recipients.

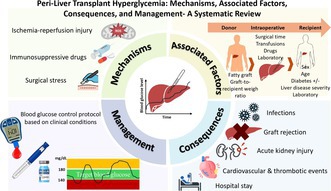

## 1 Introduction

Liver transplantation (LT) is the only effective and life‐saving treatment for patients with end‐stage liver disease (ESLD) [1, 2]. The liver is the main source of glucose synthesis and catabolism through glycogenesis, glycogenolysis, glycolysis, and gluconeogenesis. Liver transplantation is associated with various metabolic disorders. One of the most common metabolic disorders in the peri‐liver transplant period is the rapid increase in blood glucose levels after graft reperfusion [3]. This peri‐liver transplant period is defined as the time from the time of transplant surgery to the first discharge from the hospital [4]. According to the American Diabetes Association (ADA), hyperglycemia in hospitalised patients is defined as a random blood glucose level of greater than 140 mg/dL [5]. The incidence of hyperglycemia in the peri‐liver transplant period has been reported to be between 6.7% and 94% [6].

Compared with other surgical patients, liver transplant recipients encounter different challenges. LT patients often have some degree of insulin resistance due to the underlying liver diseases. In addition, the initiation of gluconeogenesis after graft reperfusion, long surgical time, and the use of high‐dose glucocorticoids and some other immunosuppressive drugs at the beginning of surgery may lead to blood glucose dysregulation [6].

Hyperglycemia following liver transplantation may be associated with an increased risk of infections (especially surgical site infections (SSIs)), graft rejection, increased length of hospital stay, and overall mortality. In addition, hyperglycemia may be associated with cardiovascular, thrombotic, or biliary complications, new‐onset diabetes after transplantation (NODAT), and acute kidney injury (AKI) [3, 4, 7, 8, 9, 10, 11, 12, 13, 14, 15, 16, 17, 18].

Several studies have compared the two methods of intensive blood glucose control and standard blood glucose control in liver transplant recipients. However, no protocol has been provided for the management of blood glucose in liver transplant recipients. Due to the high incidence of hyperglycemia after liver transplantation and its possible complications, it is important to monitor and control blood glucose levels in these patients. The purpose of this review was to elucidate the mechanisms, risk factors, and consequences of hyperglycemia and to provide some suggestions for the management of hyperglycemia immediately post‐liver transplantation period.

## 2 Methods

**FIGURE 1:**
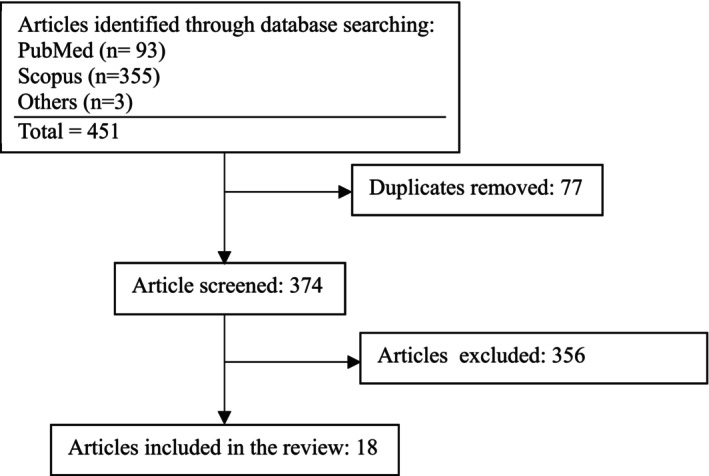
Flow diagram of the selection process.

In this comprehensive review, PubMed and Scopus databases were searched without time limitation until February 2025. All MeSH terms for “liver transplantation,” “hyperglycemia,” “blood glucose,” “risk factors,” and “postoperative complications” were used as keywords (Figure 1). The search was restricted to English‐language publications. We included all types of publications, including original studies, systematic reviews, narrative reviews, case series, and conference abstracts. Articles were excluded only if they were animal studies or not directly related to post‐transplant hyperglycemia. In addition, reputable databases (e.g., UpToDate) and diabetes guidelines were checked for detailed information on hyperglycemia management in hospitalised patients until February 2025. Finally, based on the clinical conditions of the liver transplant recipients, some suggestions are provided.

## 3 Results

The screening and selection process is shown in Figure 1. A total of 451 articles were identified, of which 374 remained after removing duplicates. After screening, 18 articles were included. The mechanisms of peri‐LT hyperglycemia, associated factors, consequences of hyperglycemia on LT outcomes, and management of peri‐LT hyperglycemia are discussed below.

### 3.1 Mechanisms of Peri‐Liver Transplant Hyperglycemia

**FIGURE 2:**
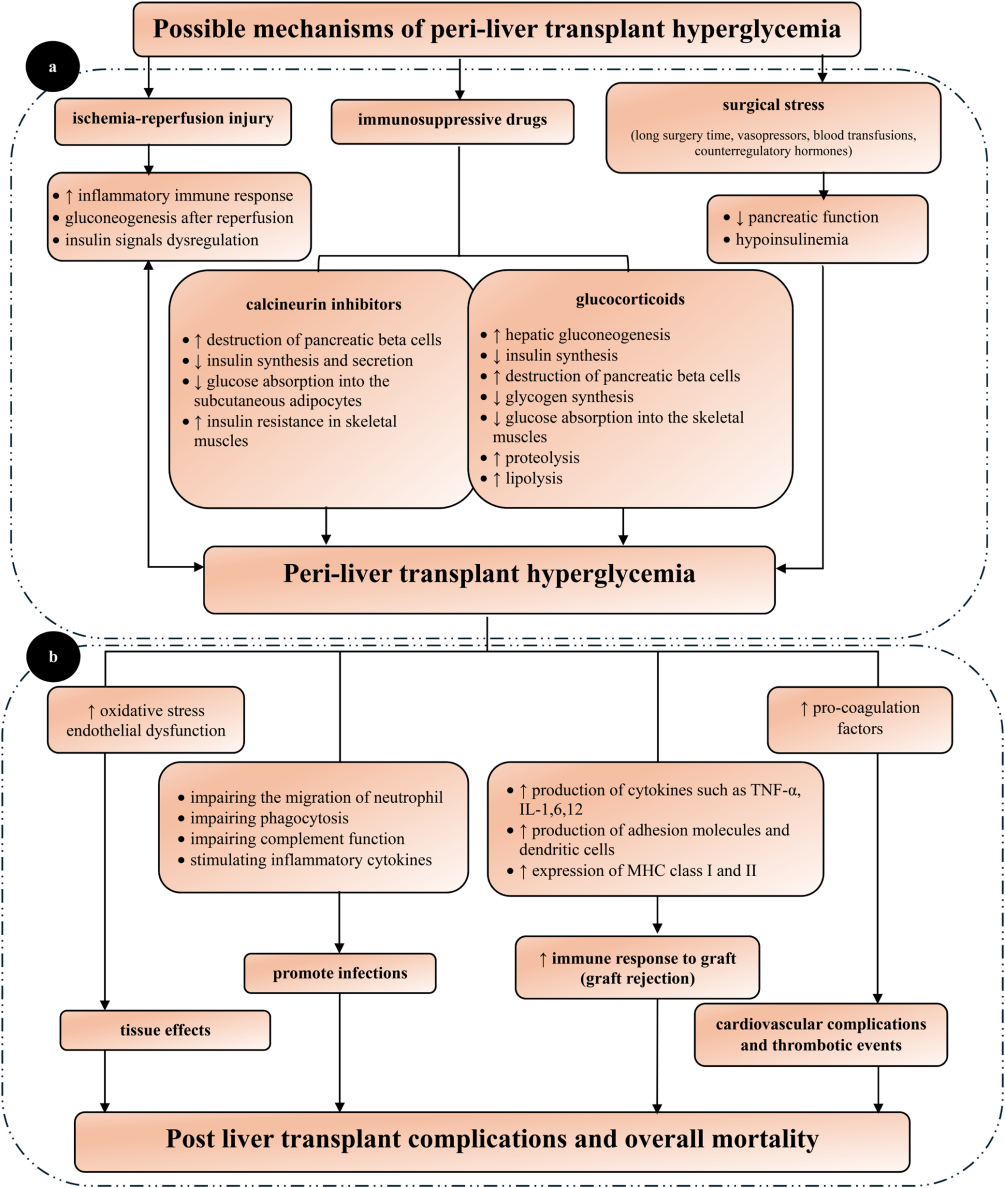
(a) Summary of possible mechanisms of peri‐liver transplant hyperglycaemia; (b) Summary of possible consequences following peri‐liver transplant hyperglycaemia.

Typically, the increasing trend of blood glucose during liver transplantation exhibits two distinct peaks. The first peak occurs during the transition from the pre‐operative phase to the pre‐anhepatic phase. This increase in blood glucose levels is related to the recipient's stress status, increased secretion of counter‐regulatory hormones, and infusion of vasopressors and blood products. The second peak occurs during the transition from the anhepatic to neo‐hepatic phase. This peak is associated with the function of the transplanted liver [19]. Each of the mechanisms is explained in detail in the relevant section and is briefly shown in Figure 2a.

#### 3.1.1 Post‐Reperfusion Hyperglycemia

Liver ischemia–reperfusion injury (IRI) following liver transplantation is a potential mechanism underlying post‐reperfusion hyperglycaemia. IRI leads to liver dysfunction and failure. The inflammatory immune response plays an important role in this phenomenon. Ischemia‐induced tissue damage causes the activation of innate immunity and the production of cytokines and chemokines. These inflammatory molecules induce more hepatocyte damage in the reperfusion phase. Finally, damage to hepatocytes increases cell membrane permeability, resulting in the release of intracellular glucose into the bloodstream [20]. Hyperglycaemia is caused by the systemic accumulation of glucose released from the destroyed hepatocytes during IRI [3]. Furthermore, based on animal studies, IRI inhibits insulin secretion and induces insulin resistance by dysregulation of insulin signals [21]. On the other hand, a transient period of hyperglycaemia aggravates IRI through oxidative stress and disruption of the tissue microcirculation [22]. Oxidative stress caused by acute hyperglycaemia is a systemic phenomenon that can affect different tissues and organs [23]. Mitochondrial structure and function of hepatocytes can be protected by controlling blood glucose levels [24]. Insulin is used both for blood glucose control and as a scavenger of free radicals released during IRI [25].

#### 3.1.2 Surgical Stress

The liver transplant surgery process is more complex and longer than most other surgeries, inducing greater surgical stress and subsequent blood glucose dysregulation [4, 12]. The surgery‐induced stress reduces pancreatic function, resulting in a decrease in the plasma insulin level. This hypoinsulinemia, along with insulin resistance and excessive catabolism due to counter‐regulatory hormones (cortisol, catecholamines, growth hormone, and glucagon), leads to hyperglycemia. Furthermore, surgery‐induced stress is aggravated by repeated blood transfusions and fluid infusion during surgery. Poor intraoperative circulatory status increases the need for exogenous catecholamines, which exacerbates hyperglycemia. In addition, the use of high‐dose glucocorticoids immediately after reperfusion in liver transplantation aggravates intraoperative hyperglycemia [26].

#### 3.1.3 Immunosuppressive Drugs

Liver transplant recipients require immunosuppressive drugs such as systemic glucocorticoids and calcineurin inhibitors (CNIs) to ensure optimal liver transplant outcomes. Nevertheless, these drug classes can negatively impact blood glucose levels [27, 28].

Liver transplant recipients receive high doses of glucocorticoids during surgery and within a few days afterward. Glucocorticoid‐induced hyperglycemia is mediated by enhancing hepatic gluconeogenesis, reducing insulin synthesis and increasing destruction of pancreatic beta cells, decreasing glycogen synthesis and glucose absorption into the skeletal muscles, and increasing proteolysis and lipolysis. Glucocorticoid‐induced hyperglycemia is dose‐dependent, meaning reducing glucocorticoid doses in the days following transplantation can mitigate their impact on blood glucose levels [27].

CNIs, the cornerstone of immunosuppressive therapy among liver transplant patients, are widely distributed in various tissues, including pancreas, skeletal muscles, heart, neurons, and adipocytes. The exact mechanism of CNI induced hyperglycemia is not known. These drugs directly disrupt the function of pancreatic beta cells and inhibit insulin synthesis and secretion, inhibit glucose absorption into the subcutaneous adipocytes, and increase insulin resistance in the skeletal muscles. Tacrolimus has more diabetogenic effects than cyclosporine [28].

### 3.2 Factors Associated With Peri‐Liver Transplant Hyperglycemia

**FIGURE 3:**
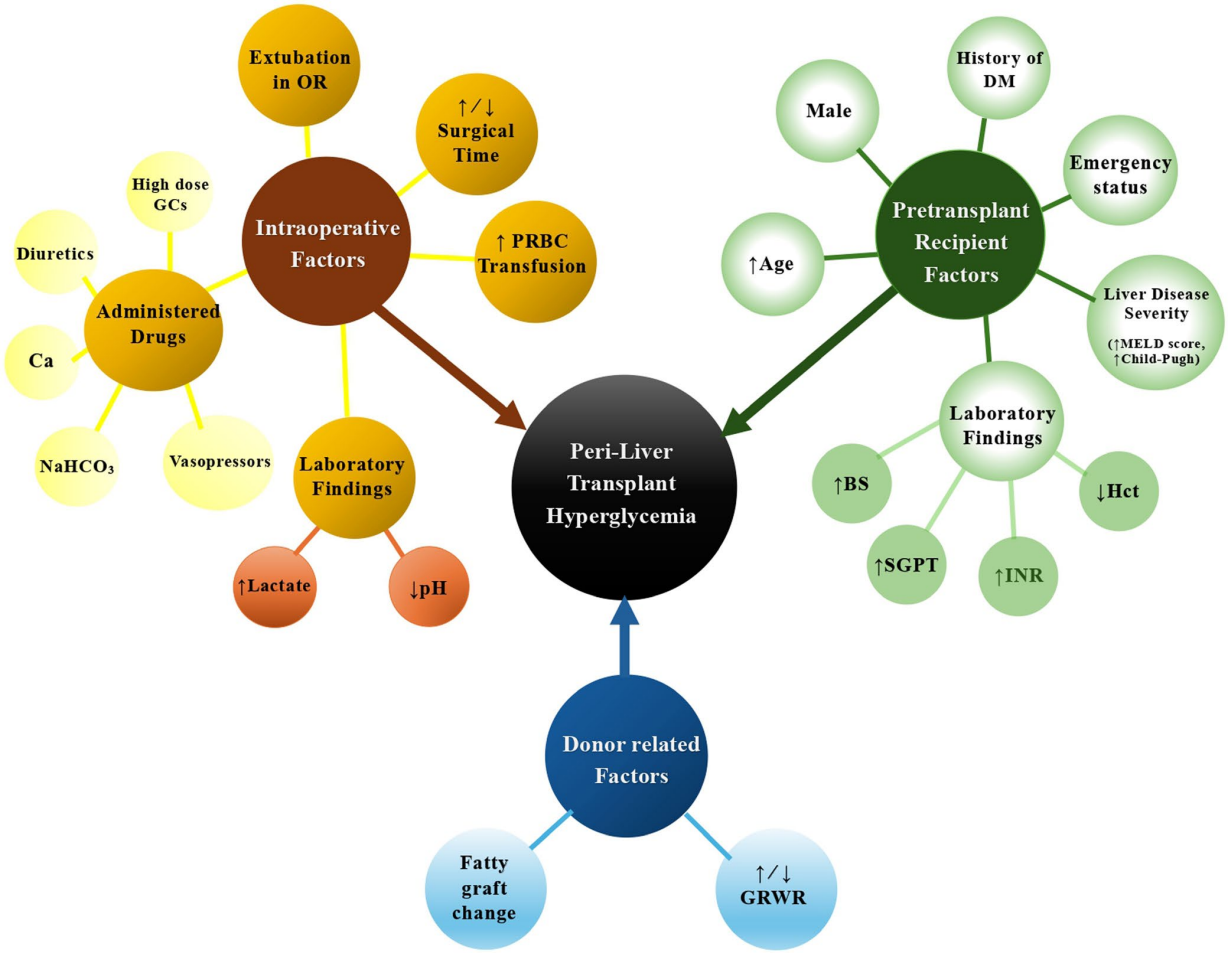
Associated factors for peri‐liver transplant hyperglycemia that reported a significant relationship in studies. BS: Blood sugar, Ca: Calcium chloride/gluconate, DM: Diabetes mellitus, GCs: Glucocorticoids, GRWR: Graft‐to‐recipient weight ratio, Hct: Haematocrit, INR: International normalised ratio, MELD: Model for end‐stage liver disease, NaHCO_3_: Sodium bicarbonate, OR: Operating room, PRBC: Packed red blood cell, SGPT: Serum glutamic‐pyruvic transaminase.

Limited studies, two [19, 29] in living and one [30] in deceased donors liver transplantation, evaluated factors that were associated with peri‐liver transplant hyperglycaemia. It seems that there is an overlap between some patients in the two studies by Chung and colleagues during 2014 and 2015 [19, 29]. These three studies are summarised in Table 1. As shown in Figure 3, these associated factors can be classified into three categories, including pretransplant recipients' factors, intraoperative factors, and donor‐related factors. These factors are discussed in more detail in the sections below.

**TABLE 1:** Detailed information regarding factors associated with hyperglycaemia in the peri‐liver transplant period.

Authors Country Year	Type of study	*n*	Group 1	Group 2	Results
Chung et al. Korea 2014 29	Retrospective	203	119 recipients: ND group	84 recipients: RH group	RH group versus ND group: Male recipients (77.4% vs. 63.9%, *p* = 0.039) MELD score (18.7 vs. 15.2 points, *p* = 0.032) Child‐Pugh class C (52.4% vs. 37%, *p* = 0.029) Emergency status (33.3% vs. 13.4%, *p* = 0.001) Levels of liver enzyme (GPT) (525.4 vs. 145.7 U/L, *p* = 0.015) Surgical time (526.6 vs. 580 min, *p* < 0.001) Level of initial lactate (3.4 vs. 2.1 mmol/L, *p* < 0.001) Level of final lactate (6.7 vs. 5.3 mmol/L, *p* = 0.004) Initial arterial pH (7.396 vs. 7.427, *p* = 0.007) Small donated grafts (GRWR) (0.46% vs. 0.5%, *p* = 0.003) Age (48.7 vs. 51.3 years, *p* = NS) BMI (24.3 vs. 24.1 kg/m^2^, *p* = NS) History of chronic underlying diseases (*p* = NS) Complications by advanced liver diseases (*p* = NS) Mean blood pressure fluctuation (13.5 vs. 13.5 mmHg, *p* = NS) Transfusion of PRBCs (10.2 vs. 10.7 pints, *p* = NS) Total amount of fluid solutions (5.69 vs. 6.15 L, *p* = NS) Administered drugs (*p* = NS)
Chung et al. Korea 2015 19	Retrospective	279	151 recipients: non‐PRSH (BG < 230 mg/dL)	128 recipients: PRSH (BG ≥ 230 mg/dL)	PRSH group versus Non‐PRSH group: History of diabetes (33.6% vs. 17.9%, *p* = 0.003) Preoperative glucose levels (128.4 vs. 112.4 mg/dL, *p* = 0.008) Preoperative haematocrit (29.1% vs. 31.3%, *p* = 0.005) Preoperative INR (1.53 vs. 1.4, *p* = 0.010) Extubation in OR (35.2% vs. 51.7%, *p* = 0.006) Transfusion of PRBCs (10 vs. 7 pints, *p* = 0.004) Furosemide administration (10 vs. 5 mg, *p* = 0.016) Calcium chloride administration (450 vs. 210 mg, *p* = 0.002) Sodium bicarbonate administration (69.8 vs. 44.2 mEq, *p* = 0.048) Inotropic score (3 vs. 0 points, *p* = 0.028) Graft fatty change (5% vs. 3%, *p* = 0.003) GRWR (1.22% vs. 1.06%, *p* < 0.001) Age (52.6 vs. 51 years, *p* = NS) BMI (24.1 vs. 24.4 kg/m^2^, *p* = NS) Male recipients (65.6% vs. 73.5%, *p* = NS) Liver disease severity (MELD score, Child‐Pugh, liver enzyme) (*p* = NS) Complications by advanced liver diseases (*p* = NS) Emergency status (14.8% vs. 15.2%, *p* = NS) Surgical time (549 min vs. 534 min, *p* = NS) Intraoperative vital sign (*p* = NS) Total amount of intraoperative fluid solutions (6.1 vs. 5.9 L, *p* = NS) Hourly urine output (1.7 vs. 1.5 mL/kg/h, *p* = NS) Intraoperative serum lactate (5.9 vs. 5.7 mmol/L, *p* = NS) Intraoperative arterial pH (7.313 vs. 7.319, *p* = NS) Total graft ischemic time (85 vs. 93 min, *p* = NS)
Milani et al. Iran 2021 30	Retrospective	100	45 recipients: non‐PRSH (BG < 230 mg/dL)	55 recipients: PRSH (BG ≥ 230 mg/dL)	PRSH group versus Non‐PRSH group: Age (49.9 vs. 39.9 years, *p* = 0.001) Transfusion of PRBCs (2.4 vs. 1.5 pints, *p* = 0.002) Sodium bicarbonate administration (139 vs. 121 mEq, *p* = 0.054) Need to vasopressors (38.2% vs. 11.1%, *p* = 0.002) Last arterial pH (7.33 vs. 7.37, *p* = 0.011) GRWR ≥ 1.1 (42% vs. 11%, *p* < 0.001) Male recipients (76.4% vs. 73.3%, *p* = NS) History of chronic underlying diseases (*p* = NS) Liver disease severity (MELD score, Child‐Pugh) (*p* = NS) Complications by advanced liver diseases (*p* = NS) Surgical time (347 min vs. 327 min, *p* = NS) Intraoperative fluid solutions (3.6 vs. 3.3 L, *p* = NS) Hourly urine output (2.4 vs. 2.8 mL/kg/h, *p* = NS) Graft fatty change (*p* = NS)

Abbreviations: BG, blood glucose; BMI, body mass index; GPT, glutamic‐pyruvic transaminase; GRWR, graft‐to‐recipient weight ratio; MELD, model for end‐stage liver disease; ND, normal decline; NS, not significant; PRBC, packed red blood cell; PRSH, post‐reperfusion severe hyperglycemia; RH, refractory hyperglycemia.

#### 3.2.1 Pretransplant Recipient's Factors

In Chung et al. study (2014), male sex was associated with a lower graft‐to‐recipient weight ratio (GRWR), a history of diabetes, a higher need for blood transfusion, and more refractory hyperglycaemia. Furthermore, in this study, patients with more severe liver disease (higher Model for End Stage Liver Disease (MELD) score or Child‐Pugh score) and emergency status, such as acute liver failure, experienced further hyperglycaemia [29]. In the other two studies, there was no correlation between hyperglycaemia and the severity of liver disease or emergency status. It may be justified that patients with a high MELD score and advanced cirrhosis have lost their glucose storage [19, 30].

Only in Chung et al.' study (2015), most recipients in the post‐reperfusion severe hyperglycemia (PRSH) group had a previous history of diabetes and higher baseline glucose levels [19]. In contrast, the other two studies found no correlation between post‐transplant hyperglycemia and underlying patient diseases [29, 30]. It seems that underlying diabetes aggravates hepatocyte damage during ischaemia–reperfusion injury due to vascular complications and hyper‐inflammatory conditions [21].

No correlation between peri‐transplant hyperglycemia and complications of advanced liver diseases (ascites, varix bleeding, hepatic encephalopathy, portopulmonary hypertension, and hepatorenal syndrome) or the cause of liver transplantation was found in available studies [19, 29, 30]. Among the pretransplant laboratory findings, lower haematocrit and higher INR were reported to be associated with severe peri‐transplant hyperglycemia [19].

#### 3.2.2 Intraoperative Factors

Although it seems that the shorter surgical time may result in less ischemia reperfusion injury and consequently lower risk of hyperglycemia [12], Chung et al. unexpectedly showed that shorter surgical time may cause delayed graft recovery and impaired glucose uptake, and subsequent higher risk of refractory hyperglycemia [29].

Chung et al. (2015) and Milani et al. found that due to the poor intraoperative circulatory status in the post‐reperfusion severe hyperglycemia group, the need for diuretics, calcium, sodium bicarbonate, and vasopressors was greater in this group [19, 30]. Also, in Chung et al.'s study (2014), circulatory insufficiency was measured by intraoperative serum lactate level and arterial pH. Poor circulatory status necessitated the increased use of exogenous catecholamines, which in turn led to more severe hyperglycemia [29].

Unlike Chung et al. report (2014), studies by Milani et al. and Chung et al. (2015) revealed that the blood transfusion rate was significantly higher in the post‐reperfusion severe hyperglycemia group [19, 29, 30]. Transfusions of packed red blood cells may lead to elevated blood glucose levels due to the glucose present in the red blood cells. Consumption of glucose‐free fluid solutions was not related to the incidence of hyperglycemia in these three studies [19, 29, 30].

In addition, a high dose of methylprednisolone appears to increase the risk of hyperglycemia at the time of reperfusion by the mechanisms described above. These studies did not mention this factor.

#### 3.2.3 Donor‐Related Factors

Chung et al. study (2014) reported that a small donated graft is associated with the risk of refractory hyperglycemia [29]. Insufficient graft size may lead to suboptimal hepatic function following transplantation. The smaller donated grafts showed impaired metabolic capacity including glucose homeostasis maintenance in the systemic circulation. Clinical evidence identifies a GRWR below 0.8% as a significant risk factor for small‐for‐size syndrome, while a minimum GRWR of 1% is generally recommended to ensure adequate functional graft mass. In contrast, Chung et al. (2015) and Milani et al. reported that a large donated graft is associated with the risk of post‐reperfusion severe hyperglycemia. A larger graft possesses greater glycogen storage capacity, which, during ischemia–reperfusion injury, can lead to increased glucose release into the systemic circulation [19, 30].

The liver is the primary organ responsible for glucose uptake when blood glucose levels rise. Following reperfusion, the transplanted liver resumes this critical function. However, any functional impairment in the transplanted liver can compromise its ability to effectively uptake glucose. Accordingly, Chung et al. found that fatty graft changes were associated with an increased risk of severe hyperglycaemia post‐reperfusion [19].

### 3.3 Possible Consequences of Peri‐Liver Transplant Hyperglycemia

#### 3.3.1 Infections

Due to the complexity of surgery and the involvement of the hepatobiliary system, liver transplant recipients are more susceptible to bacterial infections compared with other organ transplant patients [26]. Liver transplant recipients with hyperglycaemia are three times more prone to surgical site infection than recipients without hyperglycaemia [13]. The correlation between peri‐liver transplant hyperglycaemia and various major infections, such as SSIs, pneumonia, bloodstream infections, peritonitis, urinary tract infections (UTIs), and other infections, is a matter of debate.

Ammori et al. [7] and Kang et al. [3] reported that intraoperative strict blood glucose control significantly reduced the incidence of major infections after liver transplantation. Another prospective, randomised study revealed that liver transplant recipients with a target glucose level of 140 mg/dL immediately postoperatively experienced a significantly reduced risk of developing any infection within the first year [17]. In addition, Park et al. showed that intraoperative severe hyperglycemia (BG > 200 mg/dL) is an independent risk factor for postoperative SSI [13]. In contrast, other studies reported no significant differences in the infection rates among liver transplant recipients with strict versus poor blood glucose control [4, 10, 11, 14]. Furthermore, in the study by Olivera et al., although blood glucose levels were significantly lower in the intensive blood glucose control group compared with the standard control group, there was no significant difference in the risk of surgical site infections (SSIs) within 30 days between the two groups [12]. A meta‐analysis of the aforementioned studies found that the association between intensive glycemic control and a reduced risk of SSIs is uncertain [31]. These studies have been summarised in Table 2.

**TABLE 2:** Detailed information about the association between peri‐liver transplant glucose control and post‐transplant outcomes.

Authors Country Year	Type of Study	*n*	Group 1	Group 2	Study Outcomes and Results
Ammori et al. United States 2007 7	Retrospective	184	60 recipients: strict glucose control (BG < 150 mg/dL intraoperatively)	124 recipients: poor glucose control (BG > 150 mg/dL intraoperatively)	Strict versus poor glucose control group: 30‐day infections (30% vs. 48%, *p* = 0.02) 1‐year survival (91.2% vs. 78.1%, *p* = 0.05) 2‐year survival (85.9% vs. 72.7%, *p* = 0.05) Acute cellular rejection (18% vs. 12%, *p* = NS) Hepatic artery thrombosis (0% vs. 2%, *p* = NS) Biliary complications (15% vs. 19%, *p* = NS) Re‐transplantation (3% vs. 2%, *p* = NS) Myocardial infarction (2% vs. 2%, *p* = NS) AKI requiring dialysis (15% vs. 19%, *p* = NS) Venous thromboembolism (7% vs. 7%, *p* = NS)
Anderson et al. United States 2009 8	Retrospective	45	11 recipients: NODAT	34 recipients: normoglycemia	NODAT group versus normoglycemia group: Preoperative glucose (109.8 vs. 106.4 mg/dL, *p* = NS) At week 2, fasting blood glucose levels (116.4–151.8 vs. 101.7–148.4 mg/L) Acute rejection (27.3% vs. 26.5%, *p* = NS) Infection (36.4% vs. 23.6%, *p* = NS) Cardiovascular complications (27.3% vs. 5.9%, *p* = NS) Thrombotic events (2.9% vs. 11.8%, *p* = NS) Hospital readmissions (36.4% vs. 58.8%, *p* = NS)
Park et al. United States 2009 13	Retrospective	680	76 recipients: with SSI within 30 days after liver transplant	604 recipients: without SSI within 30 days after liver transplant	Independent risk factors associated with postoperative SSI: Severe (≥ 200 mg/dL), but not mild or moderate, intraoperative hyperglycemia (OR 2.25, 95% CI 1.26–4.03, *p* = 0.006) Repeat surgery (OR 6.58, 95% CI 3.41–12.69, *p* < 0.001) Intraoperative administration of vasopressor (OR 3.14, 95% CI 1.65–5.95, *p* < 0.001) Preoperative mechanical ventilation (OR 3.01, 95% CI 1.70–5.33, *p* < 0.001) Combined liver and kidney transplantation (OR 2.95, 95% CI 3.41–12.69, *p* < 0.001)
Keegan et al. United States 2010 9	Retrospective cohort	161	77 recipients: protocol group Postoperatively	84 recipients: preprotocol group postoperatively	Preprotocol group versus protocol group: Failure to reach a target glucose of < 130 mg/dL (31% vs. 6.5%, *p* < 0.01) Severe hyperglycemia (> 250 mg/dL) (15% vs. 2.8%) 1‐year mortality (OR in the protocol group 0.89, 95% CI 0.23–3.42, *p* = NS) 1‐year graft failure (OR in the protocol group 1.67, 95% CI 0.27–10.2, *p* = NS) Average time to target goal of < 130 mg/dL in protocol group: 12 h
Wallia et al. United States 2010 4	Retrospective	144	114 recipients: BG < 200 mg/dL immediate postoperative	30 recipients: BG > 200 mg/dL immediate postoperative	BG < 200 mg/dL versus BG > 200 mg/dL group: Rejection rate (35.1% vs. 76.7%, *p* < 0.001) Prolonged ventilation (32.5% vs. 13.3%, *p* = 0.047) Infection rate (59.6% vs. 53.3%, *p* = NS) Re‐hospitalisation (62.3% vs. 70%, *p* = NS) Graft survival (86% vs. 93.3%, *p* = NS) Patient survival (94.7% vs. 93.3%, *p* = NS)
Ryu et al. Korea 2013 (Abstract only) 15		326	5 groups based on their TWAG: Hypoglycemia (< 80 mg/dL) Normoglycemia (110–200 mg/dL) Mild hyperglycemia (200–250 mg/dL) Moderate hyperglycemia (250–300 mg/dL) Severe hyperglycemia (> 300 mg/dL)		AKI: compared to the normoglycemia group significantly more common in the hypoglycemia group (OR 6.768, 95% CI 1.170–39.136, *p* = 0.0327) and the severe hyperglycemia group (OR 20.702, 95% CI 3.145–136.266, *p* = 0.0016) ICU mortality: significantly higher in the hypoglycemia group compared to the normoglycemia group (OR 16.084, 95% CI 1.414–182.886, *p* = 0.0251) No significant differences between groups: Vascular complications Biliary complications SSI
Turner et al. United Kingdom 2013 (Abstract only) 16	Retrospective	58	7 recipients: NODAT	40 recipients: non‐NODAT	NODAT versus non‐NODAT group: Hyperglycemia (> 15–≤ 20 mmol/L) in the 14 days post‐transplant (5 vs. 5, *p* = 0.003) Hepatitis C (4 vs. 5, *p* = 0.018) Tacrolimus concentration (average day 1–6) (4.0 (2.6–6.6) vs. 7.0 (3.7–17.9) g/L, *p* = 0.005)
Kömürcü et al. Turkey 2015 10	Retrospective	154	58 recipients: intraoperative BG < 200 mg/dL	96 recipients: intraoperative BG > 200 mg/dL	BG < 200 mg/dL versus BG > 200 mg/dL group: Infectious complications such as: Pneumonia (15.5% vs. 13.5%, *p* = NS) UTI (10.3% vs. 13.5%, *p* = NS) SSI (3.1% vs. 3.4%, *p* = NS) Cholangitis (1.7% vs. 3.7%, *p* = NS) AKI and requiring dialysis (53.4% vs. 46.9%, *p* = 0.43 and 22.4% vs. 26%, *p* = NS) Mechanical ventilation (11.9 h vs. 22.7 h, *p* = NS) Mortality rate (31% vs. 29.2%, *p* = NS)
Yoo et al. Korea 2016 18	Retrospective, observational	304	4 groups based on TWAG: Normoglycemia (80–200 mg/dL) Mild hyperglycemia (200–250 mg/dL) Moderate hyperglycemia (250–300 mg/dL) Severe hyperglycemia (> 300 mg/dL) Also classified into quartiles depending on glucose variability (SD of glucose measurements)		No difference in the frequency (*p* = 0.072) or severity (*p* = 0.682) of AKI between the 4 TWAG groups—Compared to first quartile of variability, a greater risk of postoperative AKI in the third quartile (adjusted OR 2.47, 95% CI 1.22–5.00, *p* = 0.012) and fourth quartile (adjusted OR 2.16, 95% CI 1.05–4.42, *p* = 0.035)
Wallia et al. United States 2017 17	Prospective, Randomised	164	82 recipients: target BG level of 140 mg/dL postoperatively	82 recipients: target BG level of 180 mg/dL postoperatively	140 versus 180 mg/dL group: 1‐year infections (42.7% vs. 65.9%, *p* = 0.0046) 1‐year rejection rate (20.7% vs. 24.3%, *p* = NS)
Ramos‐prol et al. Spain 2018 14	Cross‐sectional	354	176 recipients: intensive insulin protocol postoperatively	178 recipients: conventional (sliding scale) insulin protocol Postoperatively	Intensive versus conventional insulin protocol: Rejection rate within 3 months in diabetic patients (5.5% vs. 26.5%, *p* < 0.05) Rejection rate within 5 years in diabetic patients (21.8% vs. 39.7%, *p* < 0.05) Rejection rate within 3 months and 5 years did not differ among non‐diabetic patients (*p* = NS) Mortality at 3 months (1.7% vs. 3.9%, *p* = NS) Mortality at 5 years (2.9% vs. 5.6%, *p* = NS) Infections (30.9% vs. 50.8% in diabetics and 20.7% vs. 27% in non‐diabetics, *p* = NS)
Kang et al. Korea 2018 3	Retrospective	128	89 recipients: PoIIT intraoperatively	39 recipients: CoIT intraoperatively	PoIIT versus CoIT group: Hepatocyte injury based on postoperative AST (257 IU/L vs. 390 IU/L, *p* = 0.015) Major infections (4.5% vs. 17.9%, OR = 0.22 [0.06–0.79], *p* = 0.02) Prolonged mechanical ventilation (6.7% vs. 20.5%, OR = 0.28 [0.09–0.87], *p* = 0.028) Biliary stricture (5.6% vs. 20.5%, OR = 0.23 [0.07–0.76], *p* = 0.016)
Kumar et al. United States 2020 11	Prospective, randomised	100	50 recipients: strict control group (80–120 mg/dL intraoperatively)	50 recipients: conventional control group (180–200 mg/dL intraoperatively)	Strict versus conventional control groups: Patient survival (1 year: 88% vs. 88%, *p* = NS; 3 years: 84% vs. 86%, *p* = NS; 5 years: 78% vs. 82%, *p* = NS) Graft survival (1 year: 84% vs. 88%, *p* = NS; 3 years: 76% vs. 82%, *p* = NS; 5 years: 70% vs. 78%, *p* = NS) Length of hospital stay (12.5 vs. 11 days, *p* = NS) Bile leak and biliary stricture (28% vs. 30%, *p* = NS and 26% vs. 40%, *p* = NS) CVA (0% vs. 2%, *p* = NS) Hepatic arterial stricture (2% vs. 6%, *p* = NS) Major cardiovascular event (8% vs. 10%, *p* = NS) Portal vein thrombosis (6% vs. 6%, *p* = NS) Re‐operation for bleeding (20% vs. 12%, *p* = NS) Renal failure needing dialysis (12% vs. 18%, *p* = NS) Bacterial infection (54% vs. 52%, *p* = NS) Fungal infection (22% vs. 14%, *p* = NS) Wound infection (18% vs. 14%, *p* = NS) Viral infection (8% vs. 12%, *p* = NS)
Olivera et al. Brazil 2023 12	Randomised controlled trial	41	20 recipients: IBGC (80–130 mg/dL postoperatively)	21 recipients: SBGC (< 180 mg/dL postoperatively)	IBGC versus SBGC group: Length of hospital stay (13.1 vs. 19.3 days, *p* = 0.04) 30‐day SSI (15% vs. 23.8%, *p* = NS) Time on mechanical ventilation (19.6 vs. 16.2 h, *p* = NS) Length of ICU stay (8.7 vs. 14.3 days, *p* = NS) 90‐day mortality (20% vs. 14.3%, *p* = NS)

Abbreviations: AKI, acute kidney injury; BG, blood glucose; CoIT, conventional insulin therapy; CVA, cerebrovascular accident; IBGC, intensive blood glucose control; ICU, intensive care unit; NODAT, new‐onset diabetes after transplant; NS, not significant; PoIIT, Portland intensive insulin therapy; SBGC, standard blood glucose control; SSI, surgical site infection; TWAG, time‐weighted average glucose; UTI, urinary tract infection.

Studies that identified hyperglycemia as a risk factor for various infections suggested that disruptions in the innate immune system, potentially caused by transient acute hyperglycemia or fluctuations in blood glucose levels, even over short periods, may underlie this association. This disruption is developed by impairment of neutrophil migration, phagocytosis, and complement function, as well as stimulating proinflammatory cytokines. Consequently, this impairment of the innate immune system may increase susceptibility to infections [32, 33]. Furthermore, hyperglycemia promotes the glycation of immune proteins, including immunoglobulins and collagen. The glycation of immunoglobulins results in their inactivation, while the glycation of collagen enhances collagenase activity, leading to delayed wound healing. Wound healing prolongation may increase the risk of SSIs [7].

#### 3.3.2 Graft Rejection

The correlation between perioperative glycemic control during liver transplantation and graft rejection is controversial. The main limitation for obtaining a conclusive result is the different definitions of graft rejection across various studies [4, 7, 14, 17]. In Ramos‐prol et al. study, diagnosis of graft rejection was made only by biopsy according to Banff criteria [14]; however, in Wallia et al. studies, graft rejection was defined using either clinical or pathological criteria. Clinically, rejection was identified by a twofold or greater increase in liver enzyme levels that normalised after treatment with methylprednisolone at a dose of 500 mg/day for 3 days. Pathologically, rejection was diagnosed based on the Banff criteria [4, 17]. Ammori et al. did not clarify their definitions for allograft rejection [7].

In 2010, Wallia et al. found that liver transplant recipients with a mean glucose level of less than 200 mg/dL immediately postoperatively had a significantly lower risk of rejection [4]. Similarly, a study by Ramos et al. found that diabetic liver transplant recipients who received an intensive insulin protocol had a significantly lower rejection rate within 3 months and 5 years after transplantation. In contrast, no significant difference was observed in non‐diabetic recipients [14]. In contrast, Ammori et al. found no significant difference in the acute cellular rejection rates between the two groups of liver transplant patients with strict versus poor intraoperative blood glucose control [7]. In addition, a prospective, randomised study found no correlation between maintaining target glucose levels of 140 or 180 mg/dL and the incidence of rejection within one year after liver transplantation [17]. As a secondary outcome, a meta‐analysis concluded that the relationship between intensive glycaemic control and graft rejection remains uncertain [31]. These studies have been summarised in Table 2.

Studies reporting higher rates of graft rejection in patients with suboptimal glycemic control have proposed that hyperglycemia‐induced inflammatory responses may underlie this association. Hyperglycemia stimulates the production of proinflammatory cytokines, including tumour necrosis factor‐alpha (TNF‐α), interleukins (IL‐1, IL‐6, IL‐12), adhesion molecules, and dendritic cells. Additionally, it increases the expression of major histocompatibility complex (MHC) class I and II antigens, which may enhance the immune recognition of the allograft and promote rejection [34, 35, 36].

#### 3.3.3 New‐Onset Diabetes After Transplantation (NODAT)

While peri‐liver transplant hyperglycemia is defined as a random blood glucose level greater than 140 mg/dL at the time of transplant to the first discharge from the hospital [4, 5], new‐onset diabetes after transplantation (NODAT), also known as posttransplant diabetes mellitus (PTDM), is defined as persistent hyperglycemia in recipients without a history of diabetes before transplantation and requiring antidiabetic therapy after transplantation. According to the American Diabetes Association (ADA) guideline, a fasting plasma glucose level of at least 126 mg/dL or a random plasma glucose level of 200 mg/dL or higher is defined as NODAT. Studies on the relationship between immediate hyperglycemia following liver transplantation and NODAT are scarce. NODAT is associated with various complications after transplantation, such as infections, graft rejection, and cardiovascular complications [8, 16].

In the two studies that were reviewed, it is hypothesised that recipients with poor glycemic control during the peri‐liver transplant phase may have a higher risk of NODAT within 6 months [8, 16]. In a study by Anderson et al. involving 45 liver transplant recipients, 11 individuals developed NODAT within six months post‐transplantation. While the pre‐transplant average blood glucose levels were comparable between the NODAT and normoglycemia groups, the NODAT group exhibited significantly higher glucose levels during the first two weeks following transplantation [8]. Turner et al. revealed that early hyperglycemia within the first two weeks post‐liver transplantation was associated with the development of NODAT [16]. These studies have been summarised in Table 2.

#### 3.3.4 Acute Kidney Injury (AKI)

The incidence of acute kidney injury (AKI) following liver transplantation ranges from 17% to 95%. Early postoperative hyperglycaemia through inflammatory mediators has been proposed as a modifiable risk factor for developing AKI [18, 37].

As detailed in Table 2, five studies among liver transplant patients assessed postoperative AKI and dialysis requirement as either primary or secondary outcomes. In all studies, AKI was defined based on risk, injury, failure, loss of kidney function, and end‐stage kidney disease (RIFLE) criteria [7, 10, 11, 15, 18].

Studies by Ammori et al. [7], Kumar et al. [11], and Komurcu et al. [10] found no significant differences in the incidence of acute kidney injury requiring dialysis as a secondary outcome between intraoperative strict glycemic control and poor glycemic control. In contrast, Ryu et al. reported that AKI was significantly more prevalent in the severe hyperglycemia group (BG > 300 mg/dL) than in the normoglycemia group (BG 110–200 mg/dL) [15]. Although the study by Yoo et al. did not find a significant difference in the incidence of postoperative AKI between the groups, it suggested a potential direct relationship between increased glucose variability and a higher incidence of postoperative AKI [18].

#### 3.3.5 Cardiovascular Disease (CVD) and Thrombotic Complications

Cardiovascular diseases, including peripheral arterial disease, coronary artery disease, and stroke, are significant risk factors for morbidity and mortality among organ transplant recipients. Major thrombotic events following liver transplantation include hepatic artery thrombosis, hepatic vein or inferior vena cava stenosis, venous thromboembolism, and myocardial infarction. The aetiology of these cardiovascular complications and thrombotic events in transplant recipients is multifactorial, involving the use of immunosuppressive drugs (such as glucocorticoids and calcineurin inhibitors), a history of alcohol consumption and smoking, and underlying conditions such as diabetes, hypertension, and kidney disease [38]. Few available studies have found no significant association between hyperglycemia and cardiovascular events in liver transplant recipients, suggesting that short‐term hyperglycemia may lack clinical relevance in this context [6, 7, 8, 11, 15]. These studies have been summarised in Table 2.

#### 3.3.6 Biliary Complications

The primary biliary complications following liver transplantation include biliary leaks and biliary strictures. Ischemia–reperfusion injury occurring during the peri‐transplant period, as previously discussed, can cause vascular damage to the bile ducts, leading to biliary epithelial cell injury. Hyperglycaemia may exacerbate ischemia–reperfusion injury and facilitate the infiltration of inflammatory mediators into the damaged bile ducts, potentially contributing to the development of biliary strictures. Consequently, it is hypothesized that strict blood glucose control may offer protective effects on the bile ducts [39].

As shown in Table 2, while Ammori et al. [7], Ryu et al. [15], and Kumar et al. [11] found no association between glycemic control and biliary complications, Kang and colleagues reported that the intensive insulin therapy group experienced significantly fewer biliary strictures compared with the conventional insulin therapy group [3].

#### 3.3.7 Mechanical Ventilation

The tissue effects of acute hyperglycemia are caused by an increase in inflammatory mediators and oxidative stress. One of these tissues that is affected is the diaphragm. Consequently, peri‐liver transplant hyperglycemia may contribute to prolonged mechanical ventilation as a complication. Insulin, by improving blood glucose levels and exerting a direct anabolic effect on respiratory muscles, may facilitate faster extubation of patients [23].

In Kang et al.'s study, intensive insulin therapy was significantly associated with a decreased risk of prolonged mechanical ventilation [3]. Contrary to expectations, Wallia et al. found that prolonged ventilation was significantly more common among recipients with blood glucose levels less than 200 mg/dL in the immediate postoperative period [4]. Other studies reported no significant differences between the two groups of intensive versus conventional/poor blood glucose control [10, 12]. These studies have been summarised in Table 2.

#### 3.3.8 Length of ICU/Hospital Stay

Improving blood glucose levels appears to reduce the length of ICU and hospital stays by mitigating post‐transplant complications. As detailed in Table 2, Olivera et al. found that the average hospital stay following liver transplantation was significantly shorter, by approximately 6 days, in the intensive blood glucose control group compared with the standard blood glucose control group. However, the average duration of ICU stay did not differ significantly between the two groups [12]. A study by Kumar et al. reported no significant differences in the hospital stay between liver transplant patients managed with or without intensive insulin control [11].

### 3.4 Management of Peri‐Liver Transplant Hyperglycemia

Different liver transplant centers have employed various treatment strategies for managing postoperative hyperglycemia. Most centers that reported their glycemic control strategies employed conventional methods in the control group, with the sliding scale insulin regimen being the predominant approach. These centers have introduced modified protocols to achieve strict glycemic control, comparing their clinical outcomes with conventional approaches, as summarised in Table 2. Below, we summarise the protocols used in these studies. Keegan et al. implemented a nurse‐initiated protocol. In this nurse‐initiated protocol, if the blood glucose level exceeded 130 mg/dL, a registered nurse would initiate an intravenous insulin infusion, which was then titrated hourly based on the recipient's blood glucose levels and the algorithms of the insulin infusion protocol. The protocol consisted of three tiers, with insulin infusion adjustments made according to the recipient's current blood glucose levels, the magnitude of glucose changes, and a target blood glucose range of 80–130 mg/dL [9]. A similar protocol was employed by Kumar et al. for the intraoperative strict glycemic control group. In that group, intravenous insulin infusion was initiated if the blood glucose levels exceeded 130 mg/dL or if two consecutive readings greater than 120 mg/dL were recorded within a 30‐min interval. The infusion was titrated according to defined tiers, targeting blood glucose levels between 80 and 120 mg/dL [11].

Kang et al. also evaluated the Portland protocol. The Portland protocol was initiated if, five minutes after the perfusion phase, the blood glucose level exceeded 125 mg/dL in non‐diabetic recipients, with a target blood glucose range of 80–120 mg/dL. In this protocol, both insulin bolus and infusion rates were adjusted based on blood glucose changes, previous insulin doses, and current blood glucose levels. Upon arrival in the ICU, glycemic control was managed according to a standardised protocol distinct from the intraoperative protocol. Insulin doses were increased by 2 U/h and 3 U/h for blood glucose levels of 180–240 mg/dL and 240–300 mg/dL, respectively. Conversely, insulin doses were reduced by 1 U/h and discontinued if blood glucose levels fell within the ranges of 100–140 mg/dL and below 100 mg/dL, respectively [3].

In other studies, intraoperative blood glucose management was typically overseen by the anaesthesia team, who administered intravenous insulin boluses or infusions in the intensive blood glucose control groups. Postoperatively, blood glucose control was managed by the intensive care and transplant surgery teams. However, these studies did not provide detailed tables or algorithms for these protocols [7, 12, 13].

## 4 Discussion

Hyperglycemia is common in the peri‐liver transplant period; however, studies examining the associated factors are scarce. Factors related to hyperglycemia can be categorised into three main groups: pre‐transplant recipient factors, intraoperative factors, and donor‐related factors. Pre‐transplant recipient factors, including older age, male sex, history of diabetes, severe liver disease, and emergency status, have been associated with hyperglycemia in some studies. However, other studies found no significant association, possibly due to glucose metabolism alterations in severe liver disease cases. Other recipient factors were not related to hyperglycemia or were not investigated in these studies. Intraoperative factors, including poor circulatory status, blood transfusions, and shorter surgical time, have shown varying associations with hyperglycemia. Donor factors, including graft size, have yielded conflicting results. Smaller grafts may fail to stabilise or uptake glucose effectively, while larger grafts may cause increased glucose influx during ischaemia–reperfusion injury. These discrepancies may arise from the study design limitations, small sample sizes, differences in donor types, and unmeasured variables such as the use of high‐dose glucocorticoids.

Peri‐liver transplant hyperglycemia has been linked to several complications; however, the findings across studies are inconsistent. Some studies suggest that hyperglycemia increases the risk of infections, particularly surgical site infections, potentially due to impaired immune defences and delayed wound healing. In contrast, other studies have found no significant correlation. Similarly, the relationship between hyperglycemia and graft rejection was also inconsistent. While some studies indicate that poor glycemic control may elevate graft rejection rates via inflammatory pathways, others report no significant association. The relationship between hyperglycemia and acute kidney injury also remains a topic of debate. Some studies have reported a correlation between severe hyperglycemia and increased AKI risk, while others have found no such association. Additionally, new‐onset diabetes after transplantation appears to occur more frequently in patients with elevated glucose levels shortly after transplantation, particularly when high blood glucose levels persist during the early post‐transplant period. Cardiovascular and thrombotic complications, which might be expected to be more prevalent among hyperglycemic recipients, have not demonstrated a definitive correlation in the available studies. Similarly, the evidence regarding biliary complications, mechanical ventilation, and the duration of ICU or hospital stays remains inconclusive. Although some studies suggest that strict glycemic control may mitigate these risks, others have found no significant differences between groups with varying levels of glycemic control. These varied outcomes are likely attributable to differences in study designs, glycemic control strategies and targets, sample sizes, and outcome definitions across studies. Given the aforementioned issues, it appears that effective blood glucose control in the immediate post‐liver transplantation period is crucial. Glycemic control protocols for the peri‐liver transplant period vary significantly between centres, and a comprehensive protocol has yet to be established. Importantly, many prior studies employed strict glycemic targets (blood glucose levels of 80–120 mg/dL or 80–130 mg/dL). In contrast, the current ADA guidelines recommend a target blood glucose range of 140–180 mg/dL for hospitalised patients, including critically ill patients. This discrepancy suggests that studies using stricter blood glucose targets may not reflect optimal clinical outcomes. Therefore, studies based on ADA‐recommended targets may provide better outcomes in peri‐liver transplant patients.

**FIGURE 4:**
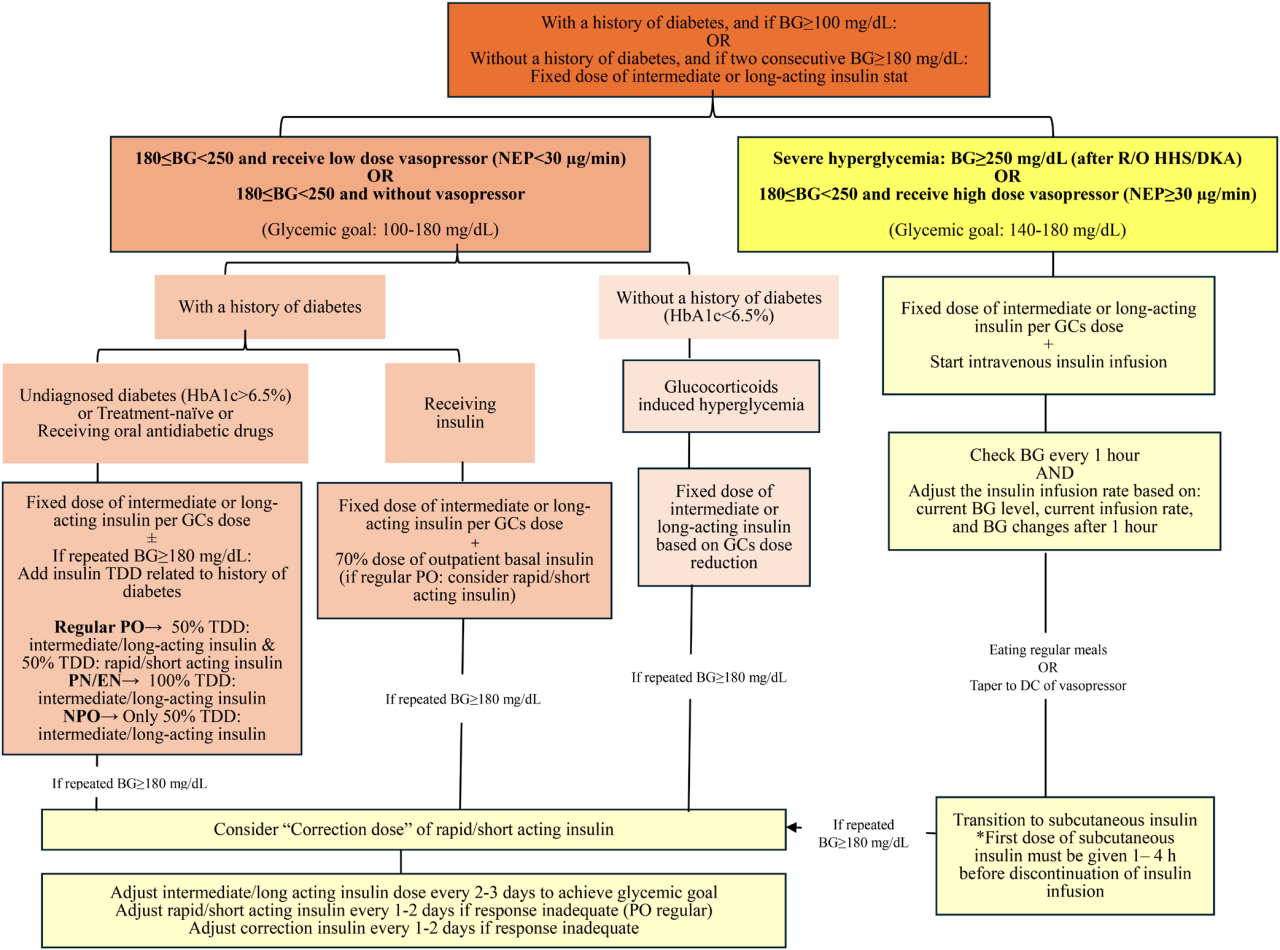
Suggested algorithm for glycemic control in liver transplant recipients; Abbreviations: BG: Blood glucose, DC: Discontinue, DKA: Diabetic ketoacidosis, EN: Enteral nutrition, GCs: Glucocorticoids, HHS: Hyperosmolar hyperglycaemic state, NEP: Norepinephrine, NPO: Nil per os, PN: Parenteral nutrition, PO: Per os, R/O: Rule out, TDD: Total daily dose.

Based on existing protocols and studies, the authors of this article propose a protocol that will be detailed in a subsequent section. As shown in Figure 4, this proposed protocol is tailored to the clinical conditions of liver transplant recipients. It is essential to note that, due to the heightened risk of AKI and hypoglycaemia in liver transplant recipients, insulin dosing should be approached with greater caution than typically recommended in standard guidelines.

According to the American Diabetes Association (ADA) guidelines, insulin therapy should be initiated in hospitalised patients with persistent hyperglycemia when blood glucose levels exceed 180 mg/dL on two occasions. Once therapy is initiated, a target glycemic range of 140–180 mg/dL is recommended for most critically ill patients with hyperglycemia [5]. The Modified Yale protocol, with a blood glucose target of 140–180 mg/dL, provides guidelines for adjusting insulin infusion rates in liver transplant recipients [40, 41]. In critically ill liver transplant recipients, research is limited. Available studies have proposed the initiation and adjustment of insulin infusion according to their respective protocols, often with a lower target blood glucose range of 80–120 mg/dL [3, 9, 11].

Based on ADA guidelines, the aforementioned studies, and the clinical situations and risks associated with liver transplant recipients, the authors of this review recommend the following blood glucose management strategy. Continuous intravenous insulin infusion is initiated for certain liver transplant recipients in critical care settings, specifically those with severe hyperglycemia (blood glucose ≥ 250 mg/dL) after excluding hyperglycemic emergencies such as diabetic ketoacidosis or hyperosmolar hyperglycemic shock. Also, in recipients with 180 ≤ blood glucose < 250 mg/dL and receiving high‐dose vasopressors (e.g., norepinephrine ≥ 30 μg/min), continuous intravenous insulin infusion is initiated because the efficacy of subcutaneous insulin is not reliable. It is suggested to consider a fixed dose of intermediate‐ or long‐acting insulin alongside intravenous insulin infusion to manage glucocorticoid‐induced hyperglycemia.

Until blood glucose levels stabilise, as indicated by three consecutive readings within the target range, patients receiving insulin infusion should have their blood glucose measured hourly. Once blood glucose levels have stabilised for 12 to 24 h, monitoring can be reduced to every 2 h. Thereafter, if there are no significant changes in nutritional intake or clinical conditions, blood glucose levels can be monitored every 4 h. However, if any of the following conditions occur—such as changes in insulin infusion rate, significant alterations in the clinical status, initiation or discontinuation of vasopressors, commencement or cessation of renal replacement therapy, or significant changes in nutritional intake—hourly blood glucose monitoring should be resumed until two to three consecutive readings fall within the target range. For critically ill patients, it is preferable to obtain blood glucose samples from indwelling vascular catheters. Blood glucose measurements using glucometers in these patients are unreliable and should be avoided whenever possible [40].

For inpatient management of hyperglycemia in noncritical care settings, a glycemic target range of 100–180 mg/dL is recommended. In patients receiving three main meals, blood glucose levels should be measured with a glucometer before each meal and at bedtime. For patients who are nil per os (NPO) or receiving enteral or parenteral nutrition, blood glucose levels should be measured with a glucometer every 4 to 6 h. If these patients regularly experience hypoglycemia, blood glucose levels should be monitored more frequently [5]. Patients without a history of diabetes (HbA1c < 6.5%) who experience hyperglycemia during hospitalisation are considered to have stress‐induced or drug‐induced hyperglycemia [42].

Insulin is the preferred choice over oral antidiabetic agents for managing glucocorticoid‐induced hyperglycemia, particularly in significant hyperglycemia and severe illness. In liver transplant recipients, methylprednisolone is predominantly used for immunosuppression. Since methylprednisolone is an intermediate‐acting glucocorticoid, the most suitable insulin for managing hyperglycemia is an intermediate‐acting basal insulin, such as NPH insulin [43]. Following liver transplantation, as the glucocorticoid dose is gradually reduced and calcineurin inhibitors are initiated alongside glucocorticoids within 1 to 5 days post‐transplant, the NPH insulin dose would be adjusted in accordance with the glucocorticoid dosage.

Liver transplant recipients with a history of diabetes can be categorised into three groups: those with undiagnosed diabetes (HbA1c > 6.5%) or treatment‐naive individuals, those with a history of insulin therapy, and those with a history of oral antidiabetic medication use. For recipients with undiagnosed diabetes or a history of oral antidiabetic medication use, a fixed dose of intermediate‐ or long‐acting insulin may initially be considered to manage glucocorticoid‐induced hyperglycaemia. If blood glucose levels remain uncontrolled, the total daily dose of insulin is calculated and initiated based on the patient's nutritional status. If further adjustments are needed due to persistent uncontrolled blood glucose levels, a correction dose of short‐ or rapid‐acting insulin may be added. For diabetic recipients with a history of insulin therapy, it is recommended to initiate 70% of their prior basal insulin dose in combination with a fixed dose of intermediate‐ or long‐acting insulin for taking glucocorticoid. If recipients take regular PO and blood glucose levels remain uncontrolled, short‐ or rapid‐acting insulin before each meal should be considered. If blood glucose levels remain uncontrolled, a correction dose of short‐ or rapid‐acting insulin should be added. After 2–3 days, the recipient's daily insulin regimen was then titrated based on the blood glucose trends and the cumulative correction insulin doses administered.

This review has several limitations that should be considered. The existing literature on associated factors and consequences reports conflicting results, particularly regarding these discrepancies. These discrepancies may stem from heterogeneous study designs, varying definitions of consequences, or differences in hyperglycaemia management protocols. The scarcity of studies on each subtopic makes it difficult to establish robust conclusions. Many studies had sample sizes or short follow‐up periods, further limiting the generalisability of findings. Future large‐scale, prospective studies are needed to clarify these relationships and establish evidence‐based management guidelines for peri‐liver transplant hyperglycaemia.

## 5 Conclusion

Hyperglycemia is a prevalent issue following liver transplantation and can lead to post‐transplant complications. Given the high incidence of hyperglycemia after liver transplantation and its potential complications, monitoring and controlling blood glucose levels in these patients is crucial. However, a standardised protocol for managing blood glucose in liver transplant recipients is currently lacking. Thus, the suggestions presented in this article, which consider the recipient's medical history and clinical conditions, can serve as a framework for healthcare providers to manage peri‐liver transplant hyperglycemia effectively.

## Author Contributions

**Yekta Rameshi:** methodology, writing – original draft, writing – review and editing, data curation, investigation. **Simin Dashti‐Khavidaki:** conceptualization, supervision, writing – review and editing, project administration, investigation. **Soghra Rabizadeh:** writing – review and editing, data curation. **Mahta Alimadadi:** data curation, writing – review and editing. **Alimohammad Moradi:** data curation, writing – review and editing. **Amir Kasraianfard:** data curation, writing – review and editing. **Ali Jafarian:** data curation, writing – review and editing. **Zahra Ahmadinejad:** data curation, writing – review and editing.

## Conflicts of Interest

The authors declare no conflicts of interest.

## Data Availability

Data sharing not applicable to this article as no datasets were generated or analysed during the current study.
